# The assessment of the prognostic value of tumor markers and cytokines as SCCAg, CYFRA 21.1, IL-6, VEGF and sTNF receptors in patients with squamous cell cervical cancer, particularly with early stage of the disease

**DOI:** 10.1007/s13277-015-3914-0

**Published:** 2015-08-20

**Authors:** Beata Kotowicz, Malgorzata Fuksiewicz, Joanna Jonska-Gmyrek, Mariusz Bidzinski, Maria Kowalska

**Affiliations:** 1 The Maria Sklodowska-Curie Memorial Cancer Centre and Institute of Oncology, Laboratory of Tumor Markers, Department of Pathology and Laboratory Diagnostics, Roentgen Street 5, 02-781 Warsaw, Poland; 2 The Maria Sklodowska-Curie Memorial Cancer Centre and Institute of Oncology, Department of Urooncology, Roentgen Street 5, 02-781 Warsaw, Poland; 3 Independent Public Health Care, Inflancka Street 6, Warsaw, Poland

**Keywords:** Cervical cancer, VEGF, IL6, CYFRA 21.1, SCCAg

## Abstract

The aim of this study is to determine the prognostic value of tumor markers, as squamous cell carcinoma antigen (SCCAg) and cytokeratin-19 fragment (CYFRA 21.1) and interleukin 6 (IL-6), vascular endothelial growth factor (VEGF), soluble tumor necrosis factor receptor I (sTNF RI), and sTNF RII in patients with squamous cell carcinoma of the cervix. The subjects of analysis were 138 patients with stage I–IVA according to the International Federation of Gynecology and Obstetrics (FIGO) classification. The collected research material comes from one oncology center. During the 10 years of follow-up, 56 relapses and 53 deaths were observed, and recurrent disease in early stage was confirmed in 45 % of patients. The pretreatment serum levels of SCCAg and CYFRA 21.1, and cytokines IL-6, VEGF, sTNF RI, and sTNF RII were determined in all patients. The probability of disease-free survival (DFS) and overall survival (OS) was evaluated using the log-rank test and the Cox regression model. Based on the ROC curve analysis for patients with recurrence, the largest area under the curve was demonstrated for SCCAg and IL-6 and for patients who died, for SCCAg and VEGF. Cox analysis demonstrated that independent prognostic factor for DFS was only SCCAg and for OS cytokine IL-6 and SCCAg, but in patients with early stage the prognostic value for DFS was VEGF, whereas IL-6 and CYFRA 21.1 for OS. Serum level of VEGF, CYFRA 21.1 and IL-6 before treatment in patients with early stage cervical cancer appears to be an important prognostic factor.

## Introduction

Due to well-developed screening programs, the incidence of cervical cancer, especially squamous cell carcinoma (SCC), has decreased dramatically, although cervical cancer is still the third most diagnosed cancer and the fourth highest cause of death among females around the world [1].

Some pathologic features, such as tumor diameter, lymph node involvement, lymph vascular space invasion, extend of the tumor, and depth of stromal invasion, have been proven as clinically important [2]. Optimal management consists of appropriate treatment methods after the precise staging, following detection as early as possible, and the best salvage therapy. Early detection of recurrence, especially at the early stages in cervical cancer patients, is of main importance. The survival improvement from early detection of disease relapse has been found [3, 4]. So far, several methods are available for recurrence detection, but the disease is usually advanced with the accompanying clinical symptoms. The main problem is detection of the disease in the preclinical phase, with no visible lesions in computed tomography (CT) or magnetic resonance (MRI), while the chance for a cure is the highest. Several tumor markers have been investigated so far, in which the most commonly used is squamous cell carcinoma antigen (SCCAg). In studies presented by Scambia et al. and Strausss et al., 74–88 % of patients presented an elevation of SCCAg with association of disease, while 70–86 % of recurred patients were proven to have elevated SCCAg [5, 6]. In some cases, SCCAg is not elevated in disease recurrence or at the initial diagnosis [5]. Investigation of the most sensitive useful marker to detect disease recurrence at the earliest stage is needed.

One of the most promising appears to be serum cytokines produced by a wide variety of cells, mainly monocytes and macrophages, which plays a central role in the regulation of the inflammatory and immune system [7]. The correlation of cytokines with disease severity and survival has been studied to date in breast cancer, ovarian cancer, as well as cervical cancer [8–13].

The aim of the study was to determine the prognostic value of pretreatment serum levels of the cytokines interleukin 6 (IL-6), vascular endothelial growth factor (VEGF), soluble tumor necrosis factor receptor (sTNF RI) and sTNF RII in patients with SCC of the cervix and the tumor markers SCCAg and cytokeratin-19 fragment (CYFRA 21.1).

## Materials and test methods

During the period from 2005 to 2008, 138 cervical cancer patients (ages 26–85; median 54 years) with stage I–IVA, according to the International Federation of Gynecology and Obstetrics (FIGO) classification, were treated at the Maria Sklodowska-Curie Memorial Cancer Centre and Institute of Oncology in Warsaw. Written informed consent from all patients and the approval of the local Ethical Committee Nr 26/2005 was obtained before treatment. The workup for all patients performed before treatment included clinical examination, computed tomography (CT) or magnetic resonance imaging (MRI), chest X-ray, blood count, and biochemistry. The pretreatment serum levels of the tumor markers SCCAg and CYFRA 21.1, and certain cytokines, such as IL-6, VEGF, sTNF RI, and sTNF RII, was determined for all patients.

SCCAg was determined by chemiluminescence (CMEIA) from Architect i2000sr kits, Abbott Diagnostics, CYFRA 21.1 was determined by electrochemiluminescence using Cobas system 6000 sets from Roche, and cytokine concentrations were determined in serum using ELISA kits from R&D Systems.

Clinicopathological features of patients with squamous cell carcinoma of the cervix, taking into account the clinical condition, are presented in Table 1. In patients treated surgically, the assessment of lymph nodes was performed during the histological assessment of the surgical specimen. In patients treated with radiochemotherapy (CCR), the assessment of lymph nodes was performed using the imaging studies as MRI or CT. Twenty-nine patients (20.9 %) underwent the surgical treatment. Among this group, nine (6.5 %) patients were treated with radical hysterectomy (RH) with no treatment afterwards, ten (7.2 %) with RH and brachytherapy (BT), and ten (7.2 %) with RH and CCR. 109 (79.1 %) were treated with CCR alone. The SC type of the tumor was confirmed histologically for all patients before treatment. CCR consisted of irradiation with high megavoltage photons, 15 MeV X-rays, from a linear accelerator, and chemotherapy. External beam radiotherapy (EBRT) was administered in daily fractions of 1.8–2 Gy up to 45–50 Gy. Concomitant chemotherapy (CHT) consisted of once-weekly infusion of cisplatin (40 mg/m^2^ dose) with appropriate hydration. During the first 2 years, patients were followed every 3 months and twice per year thereafter. In cases of suspicion of recurrence, biopsies based on CT or MRI were obtained. In patients with SCC of the cervix, the pretreatment serum levels of cytokines and tumor markers were determined in relation to the clinical condition, and assessed after approximately 10 years of follow-up.

**Table 1:** Clinicopathological features of patients with squamous cell carcinoma of the cervix, taking into account the clinical condition

Characteristics	Patients, *n* = 138		Patients with no recurrence, *n* = 82		Patients with relapse, *n* = 56		Deaths, *n* = 53	
	*N*	%	*N*	%	*N*	%	*N*	%
Median age (range, years)	54 (26–85)		54 (28–84)		54 (26–85)		54 (26–85)	
Menopausal status								
Premenopausal	47	34	28	34	19	34	20	34
Postmenopausal	91	66	54	66	37	66	38	66
FIGO stage								
I	38	28	34	41	4	7	4	7
II	40	29	22	27	18	32	16	30
III	57	41	26	31	31	55	31	58
IV	3	2	0	0	3	5	2	4
Histological grade/G/								
G1	8	6	7	11	1	2	1	2
G2	61	44	41	63	20	47	18	34
G3	39	28	16	25	22	51	21	40
Gx	30	22	17	21	13	23	13	25
Lymph node status								
Negative	105	78	67	83	38	70	33	62
Positive	30	22	14	17	16	29	18	34
Not evaluable	3	2	1	1	2	4	2	4
Median SCCAg (ng/mL)	2.9		1.6		6.7		6.2	

*N* number, *FIGO* International Federation of Gynecology and Obstetrics, *SCCAg* squamous cell carcinoma antigen

The reference group consisted of 50 healthy people in our earlier study, designated as cutoff value for the cytokines IL-6, VEGF, sTNF RI, sTNF RII, assuming the 95th percentile (14). For statistical calculations, Statistica PL 6.0 for Windows and Excel 7.0 for Windows were used. For the comparison of two independent groups, a nonparametric *U* test, the Mann-Whitney test, was adopted. The probability of disease-free time (DFS) and overall survival (OS) was evaluated with univariate analysis using the log-rank test and the Cox regression model. The cutoff level of significance was set at *P* < 0.05. The analysis of the diagnostic power of determined parameters was made using the MedCalc statistical program, which included the determination of the areas under the receiver operating characteristics (ROC) curves (AUC), interdependencies AUC of determined parameters, and the determination of cutoff points for the studied biomarkers, with the optimal sensitivity and specificity of the tests.

## Results

Clinical observation of the analyzed patients was conducted until the end of 2013. During approximately 10 years of follow-up (median, 1572 days; range, 102–3375), disease relapse was observed in 56 patients (41 %), and there were 58 (42 %) deaths, including 5 deaths not related to the disease. The median time to relapse was 375 days.

In 82 patients with no recurrence confirmed clinically, the majority of determined parameters levels were elevated in 50 % of patients, with the exception of CYFRA 21.1 and the soluble receptor sTNFR II, while their median concentrations (with the exception of IL-6 and sTNF RI) were below the accepted cutoff points.

In the group of 56 patients with confirmed recurrence, the percentages of elevated concentrations and the median of all biomarkers determined were significantly higher, with the most frequently observed elevated levels from sTNF RI (96 %), SCCAg, and IL-6 (84 %). The Mann-Whitney test demonstrated that, in patients for whom recurrence of the disease was confirmed, the concentrations of all the examined parameters before treatment were significantly higher compared to the levels in patients in remission (Table 2).

**Table 2:** Median value, concentration ranges, and the percentage of patients with elevated levels of tumor markers and cytokines, and significant differences depending on the clinical condition

Markers/ Cytokines	Patients with remission, *n* = 82		Patients with recurrence, *n* = 56		Remission vs. recurrence
	Median range	%	Median range	%	*P* value
SCC Ag (ng/mL)	1.6 (0.2–54.6)	50	6.6 (0.2–299)	84	0.001
CYFRA 21.1 (ng/mL)	1.85 (0.3–105.7)	26	3.5 (0.5–62)	52	0.001
IL-6 (pg/mL)	2.6 (0.7–237)	54	5.5 (0.7–119.5)	84	0.001
VEGF (pg/mL)	325 (51.5–1401)	50	396 (99–2000)	63	0.045
sTNF RI (pg/mL)	1525 (815–2600	72	2140 (1015–5000)	96	0.001
sTNF RII (pg/mL)	2492 (1577–5000)	23	2817 (1155–5000)	60	0.006

*SCCAg* squamous cell carcinoma antigen, *CYFRA 21.1* cytokeratin fragment detected by antibodies BM 19–21 and KS 19, *IL-6* interleukin 6, *VEGF* vascular endothelial grown factor, *sTNFRI* soluble tumor necrosis factors receptor I, *sTNFRII* soluble tumor necrosis factors receptor II

**Fig. 1:**
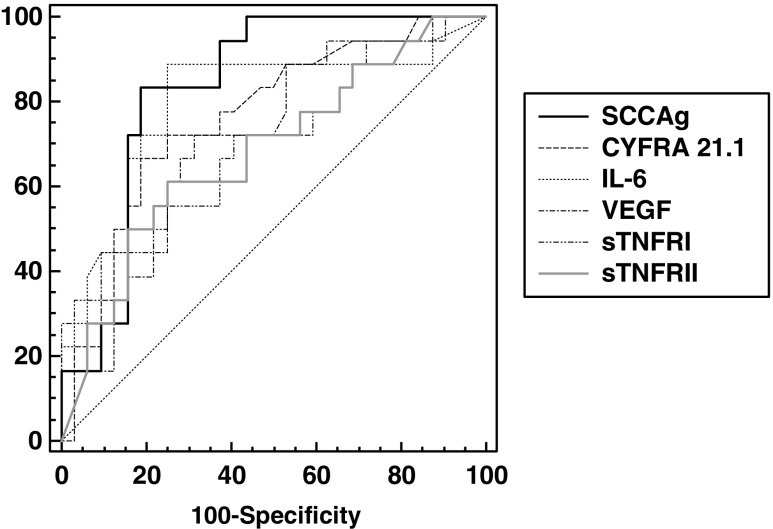
Receiver operating characteristic curves for SCCAg, CYFRA 21.1, and cytokines in cervical cancer patients with recurrence

Based on the ROC curve analysis carried out in relapsed patients versus patients in remission, the largest area under the curve (AUC) was demonstrated for SCCAg (0.833) and IL-6 (0.799). For other determined biomarkers, AUCs were CYFRA 21.1, 0.763; VEGF, 0.711; sTNF RI, 0.701; and sTNF RII, 0.682 (Fig. 1). No significant differences were found between the fields under the ROC curves for any of the parameters studied.

**Fig. 2:**
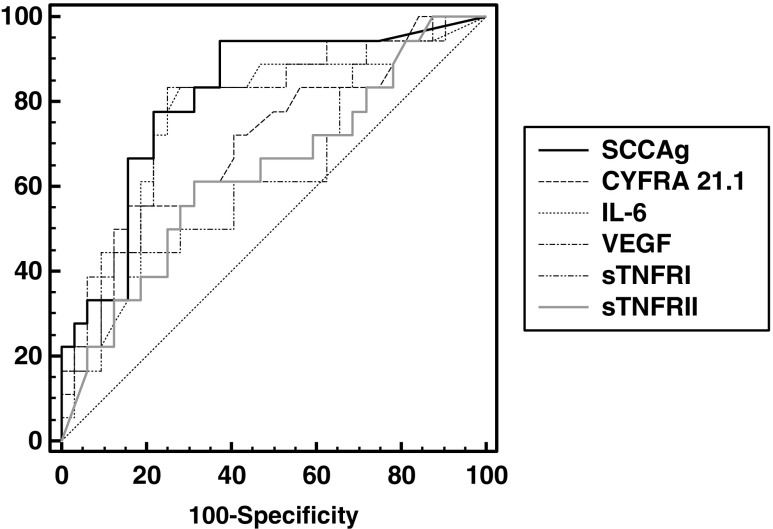
Receiver operating characteristic curves for SCCAg, CYFRA 21.1, and cytokines in cervical cancer patients who had died

In the group of 53 patients who died, the percentages of elevated concentrations of the studied parameters and their medians were similar to those in patients who relapsed. In patients whose observation ended in death, the most clinical utility was demonstrated for SCCAg (AUC = 0.811), VEGF (AUC = 0.783), and IL-6 (AUC = 0.754). For the other parameters, AUCs were CYFRA 21.1, 0.706; sTNF RI, 0.644; and sTNF RII, 0.634 (Fig. 2). In the ROC curve analysis also, significant differences between the AUC for SCCAg and sTNF RI (*P <* 0.044), and SCCAg and sTNF RII (*P <* 0.039) were confirmed. Furthermore, the Mann-Whitney test demonstrated that, in these patients, the designated concentration of all tumor markers and cytokines were significantly higher (*P* < 0.01) compared with their concentration in patients who were still alive.

Then, evaluation of the dependence between the concentrations of determined parameters before treatment and the DFS and OS was performed. Based on the ROC curve analysis for the determined biomarkers, the other cut-points were set, while maintaining optimal sensitivity and specificity of the test for groups with recurrence versus no recurrence and death versus being alive (Table 3). Relapse was observed even in patients with early disease, i.e., 11 % (4/38) in patients in FIGO stage I and 45 % (18/40) in FIGO II, and in the advanced stages, 54 % (31/57) in FIGO III and in all patients in stage IV. Relapse occurred in the highest proportion of patients with histological grade G3, 56 % (22/39). With G2 and G1, it was in 33 % (20/61) and 13 % (1/8), respectively. Among patients for whom regional lymph nodes were assessed as free, recurrence was observed in 36 % (38/105), while for patients with positive nodes, recurrence was observed in 53 % (16/30).

**Table 3:** Tumor marker and cytokine test characteristics for remission or recurrence for cervical cancer patients alive or have died

Markers/cytokines	Patients remission/recurrent				Patients alive/died			
	Cutoff	Sensitivity	Specificity	*P* value	Cutoff	Sensitivity	Specificity	*P* value
SCCAg (ng/mL)	1.8	82.1	54.9	0.001	1.6	81.0	50.0	0.001
CYFRA 21.1 (ng/mL)	2.6	65.2	68.2	0.001	3.5	51.1	80.0	0.001
IL-6 (pg/mL)	3.4	83.7	63.3	0.001	3.4	83.0	65.3	0.001
VEGF (pg/mL)	352	60.4	61.1	0.037	352	62.8	63.8	0.007
sTNF RI (pg/mL)	1540	81.8	55.3	0.001	2095	51.6	83.7	0.004
sTNF RII (pg/mL)	3500	55.0	85.7	0.002	2984	65.0	68.6	0.019

*SCCAg* squamous cell carcinoma antigen, *CYFRA 21.1* cytokeratin fragment detected by antibodies BM 19–21 and KS 19, *IL-6* interleukin 6, *VEGF* vascular endothelial grown factor, *sTNFRI* soluble tumor necrosis factors receptor I, *sTNFRII* soluble tumor necrosis factors receptor II

**Fig. 3:**
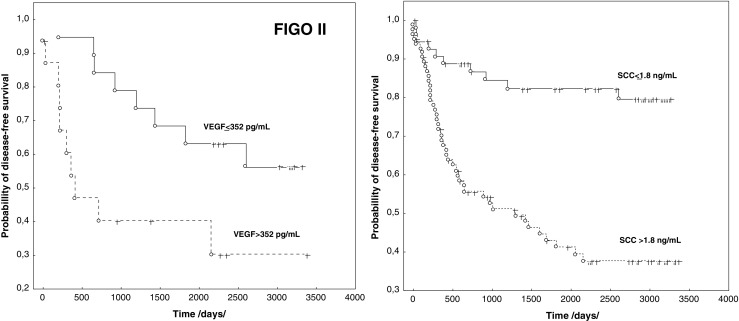
Disease free survival and VEGF concentrations in FIGO stage II and SCCAg concentrations in the entire group of patients

Because FIGO stage II recurrent disease was confirmed in 45 % of patients, the survival analysis in this group was performed. The log-rank test demonstrated a correlation between VEGF and the age of the patients with DFS, while multivariate Cox analysis found that only VEGF is an independent prognostic factor in patients in stage FIGO II (*P* < 0.016 (HR 3.559; 95 % CI 2.526–4.592). Assessing the relationship between the concentrations of the determined parameters before treatment in the entire study group and DFS in univariate analysis and the log-rank test, in addition to the FIGO stage, histological grade (G), and lymph node status, confirmed the relationship between the concentration of the tumor markers SCCAg and CYFRA 21.1, and the assayed cytokines and their receptors IL-6, VEGF, sTNFR I, sTNF RII, and DFS. However, in a Cox multivariate analysis, the prognostic value for DFS was confirmed for the FIGO stage, *P* < 0.023 (HR 1.620; 95 % CI 1.204–2.035), and G, *P* < 0.031 (HR 2.195; 95 % CI 1.485–2.905). Among the studied parameters the statistically significant influence on DFS was ascertained for SCCAg, *P* < 0.012 (HR 2.649; 95 % CI 1.890–3.408) (Fig. 3).

**Fig. 4:**
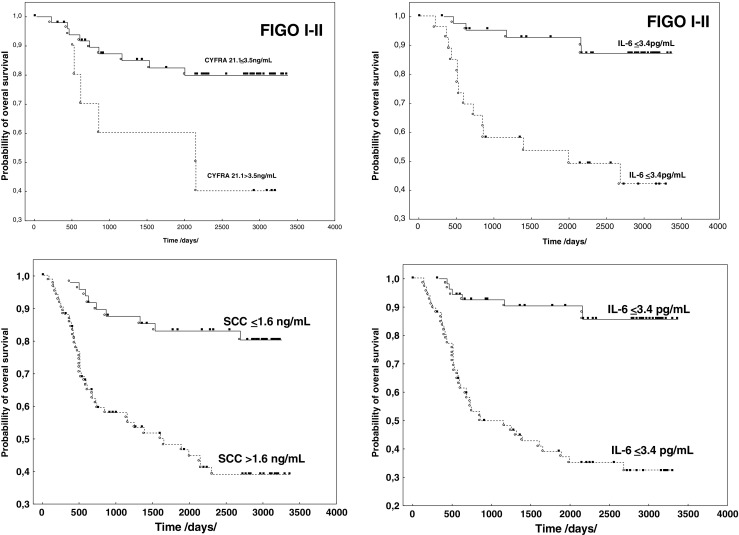
The relationship between overall survival, CYFRA 21.1, and IL-6 in FIGO stages I-II, SCCAg and IL-6 in the entire group of patients

Of the 138 patients enrolled in the study, after approximately 10 years of follow-up, 53 died from cancer, including 50 patients with confirmation of earlier disease recurrence. The median follow-up time from recurrence to death in this group was 234 days. Patients in the early stages (FIGO I-II) accounted for 36 %. Univariate analysis carried out in these patients showed a significant relationship between the concentrations of SCCAg (*P* < 0.005), CYFRA 21.1 (*P* < 0.011), IL-6 (*P* < 0.0001), VEGF (*P* < 0.004), and OS. For these parameters, the prognostic value for OS of the patients in the early stages was confirmed in the Cox analysis for concentrations of IL-6, *P* < 0.003 (HR 6.449; 95 % CI 5.212–7.686), and CYFRA 21.1, *P* < 0.009 (HR 5.192; 95 % CI 3.959–6.425). Assessing the prognostic value of tumor markers and cytokines in the entire study group log-rank test showed a significant relationship between the concentrations of all biomarkers and OS. However, in the multivariate Cox analysis, the prognostic value for OS of patients with SCC of the cervix was confirmed only for concentrations of SCCAg, *P* < 0.027 (HR 2.512; 95 % CI 1.697–3.327), and IL-6, *P* < 0.003 (HR 3.768; 95 % CI 2.892–4.644) (Fig. 4).

## Discussion

Cervical cancer is a malignancy of the reproductive tract derived from a variety of cell types. Cancer derived from squamous cells occurs more frequently and has a lower aggressiveness; however, it happens that, despite the early stage of the disease, recurrences are observed.

Therefore, looking for factors that at the time of diagnosis would be helpful in predicting its course and thus the selection of the optimal treatment regimen is of main importance. In the presented studies, the possibility of using concentrations of standard tumor markers for SCC measured before treatment was evaluated. Tumor markers SCCAg and CYFRA 21.1, cytokines IL-6 and VEGF, and soluble tumor necrosis factor receptors sTNFR I and II were evaluated as possible prognostic factors in all patients in the early stages of cancer development with a similar prognosis. Within the group of patients who, during a 10-year period of observation, have had recurrence of the disease, concentrations of all markers were significantly higher compared to concentrations noted in patients who were in remission. Furthermore, in cases of patients with progression, the highest diagnostic sensitivity was noted for concentrations of SCCAg and IL-6. In the group of patients who died, the highest diagnostic sensitivity was noted for VEGF, as well as SCCAg and IL-6. Hoogendam et al. evaluated the utility of various biomarkers in the detection of cervical cancer. They proved that SCCAg and high-sensitivity C-reactive protein (hsCRP) have the highest diagnostic sensitivity. In the cases of VEGF and IL-6, similar results were not confirmed [13]. In the next step of our studies, we determined the cutoff points for all parameters based on the ROC curve, while maintaining the optimum sensitivity and specificity of the tests. From this, we conducted an analysis of patient survival rate, searching for prognostic factors for both DFS and OS. Similar studies have been carried out by other authors, who analyzed the optimum cutoff points for biomarkers determined before beginning treatment, taking into consideration the patients’ clinical condition. Adopting individual cutoff points based on the ROC curve is important because it can increase sensitivity. This approach, in turn, may increase the chance of detecting precancerous states and enable earlier detection of recurrence [13–17].

As is known, the classic and widely recognized prognostic factor in patients with malignancy is the degree of development. Similar to our research, other authors have observed that the risk of recurrence of cervical carcinoma depends on the disease stage. Recurrence of the cancer is observed quite often. The consequences of the process are deaths [13, 15]. However, in our studied group, recurrence had already been observed in 45 % of patients in FIGO II stage. Because of this, we searched for additional prognostic factors in this specific group of patients.

The research proved that increased concentration of the proangiogenic cytokine VEGF is an independent, and at the same time poor, prognostic factor for DFS In patients in stage II cancer development. VEGF plays a specific role in the process of neoangiogenesis, which is in the progression of the disease and metastasis. The role of angiogenesis in the progression of cancer is very well documented [18–22]. Researchers found that VEGF concentrations are connected with clinical stage and size of the tumor, as well as their usefulness in monitoring the course of disease [19, 23]. Zustrzeel et al. stated that marking VEGF pretreatment concentrations in cervical cancer patients can be useful in determining DFS and OS. The dependence of VEGF on DFS and OS was confirmed in multivariate analysis using the Cox model. However, the results of the analysis concerned the entire studied group, which included adenocarcinoma and adenosquamous patients [14]. Although other authors have confirmed the participation of VEGF in the progression of cervical cancer, they did not indicate its predictive value [24]. Our studies have demonstrated the prognostic value of VEGF for DFS in patients with FIGO stage II SCC. Therefore, it appears that the relationship we have established between VEGF concentrations and DFS in patients in FIGO stage II may prove to be useful in clinical practice.

A similar analysis of OS in the first stage was conducted in patients in stage FIGO I + II. The analysis demonstrated the important role of proinflammatory cytokines IL-6 and CYFRA 21.1 as independent prognostic factors. IL-6 is a pleiotropic cytokine produced by various cells, mainly monocytes and macrophages. It plays a key role in the regulation of inflammation and immune responses, and in the pathogenesis and progression of cancer [8, 12, 25, 26].

Earlier studies have shown that IL-6 concentrations increase with the development of clinical stages in patients with cervical cancer. Complimentary marking of IL-6 with the standard tumor marker SCCAg, especially for patients in the early stages of clinical development, increases its diagnostic sensitivity [25]. In recent years, authors say that marking IL-6 concentrations before treatment can influence the estimation of DFS and OS. They place stress on the important role of proinflammatory cytokines, IL-6 included, in the progression of cervical cancer, particularly in early stages of development [5, 27]. Our studies confirmed those reports. Furthermore, the studies showed the important role of IL-6 on DFS and OS, not only for patients in FIGO stages I-II, but for the entire studied group (FIGO I-IV). Other researchers have not demonstrated such dependence when analyzing a comparable group. However, their observation period was considerably shorter [28].

CYFRA 21.1 is a cytokeratin-19 fragment that is soluble in serum. Elevated concentrations of this marker were found in a large percentage of SCC patients with different localizations of the tumor [25, 29, 30]. In cervical cancer patients, concentrations of CYFRA 21.1 increase with the progression of the disease. The literature emphasizes that this marker has a lower diagnostic utility compared to the value of SCCAg, suggesting complimentary utility of marking CYFRA 21.1 with SCCAg and other biomarkers [31, 32]. Some authors claim that, in patients in the early stages of cancer development, determining CYFRA 21.1 before beginning treatment might have prognostic value [28, 33]. Our studies have confirmed that CYFRA 21.1 has a prognostic value in connection to OS in patients with stage FIGO I-II.

In the presented study, a shorter period of survival was observed in patients with recurrence and elevated SCCAg concentrations when compared to patients with recurrence having normal SCCAg concentrations. Like other authors, we have confirmed the prognostic value of the standard SCCAg marker for both DFS and OS. This is true for the whole of the studied group irrespective of the patients’ FIGO stage [15, 16, 34]. Although the results of our study and the results presented in the works of other authors demonstrate the important prognostic value of SCCAg in the early stages of cancer development, researchers attempt marking SCCAg with other biomarkers, which is important in cases where the marker is not elevated [13, 34–36].

The assessment of the correlation of tumor markers with the response to any treatment might be also interesting, but due to relatively small number of patients in each treatment group we did not perform such analysis. The limitations in our study are the heterogeneous study population, relatively small sample size, but this is the single institution experience.

In conclusion, our study illustrated that elevated VEGF and CYFRA 21.1 concentrations before beginning treatment in patients with SCC of the cervix at the earlier stages of clinical development can be poor prognostic factors. Moreover, we demonstrated that in patients with SCCAg concentration levels that are not increased before beginning treatment, measuring interleukin 6 may be helpful in the prognosis of the disease.
